# A Heat Shock Protein 48 (HSP48) Biomolecular Condensate Is Induced during Dictyostelium discoideum Development

**DOI:** 10.1128/mSphere.00314-19

**Published:** 2019-06-19

**Authors:** Stephanie Santarriaga, Alicia Fikejs, Jamie Scaglione, K. Matthew Scaglione

**Affiliations:** a Department of Biochemistry, Medical College of Wisconsin, Milwaukee, Wisconsin, USA; b Department of Computational and Physical Sciences, Carroll University, Waukesha, Wisconsin, USA; Carnegie Mellon University

**Keywords:** *Dictyostelium discoideum*, chaperone, phase separation, small heat shock protein

## Abstract

The social amoeba Dictyostelium discoideum’s proteome contains a vast array of simple sequence repeats, providing a unique model to investigate proteostasis. Upon conditions of cellular stress, D. discoideum undergoes a developmental process, transitioning from a unicellular amoeba to a multicellular fruiting body. Little is known about how proteostasis is maintained during D. discoideum’s developmental process. Here, we have identified a novel α-crystallin domain-containing protein, heat shock protein 48 (HSP48), that is upregulated during D. discoideum development. HSP48 functions in part by forming a biomolecular condensate via its highly positively charged intrinsically disordered carboxy terminus. In addition to HSP48, the highly negatively charged primordial chaperone polyphosphate is also upregulated during D. discoideum development, and polyphosphate functions to stabilize HSP48. Upon germination, levels of both HSP48 and polyphosphate dramatically decrease, consistent with a role for HSP48 and polyphosphate during development. Together, our data demonstrate that HSP48 is strongly induced during Dictyostelium discoideum development. We also demonstrate that HSP48 forms a biomolecular condensate and that polyphosphate is necessary to stabilize the HSP48 biomolecular condensate.

**IMPORTANCE** During cellular stress, many microbes undergo a transition to a dormant state. This includes the social amoeba Dictyostelium discoideum that transitions from a unicellular amoeba to a multicellular fruiting body upon starvation. In this work, we identify heat shock protein 48 (HSP48) as a chaperone that is induced during development. We also show that HSP48 forms a biomolecular condensate and is stabilized by polyphosphate. The findings here identify Dictyostelium discoideum as a novel microbe to investigate protein quality control pathways during the transition to dormancy.

## Abstract (precis)

During cellular stress, many microbes undergo a transition to a dormant state. This includes the social amoeba Dictyostelium discoideum that transitions from a unicellular amoeba to a multicellular fruiting body upon starvation. In this work, we identify heat shock protein 48 (HSP48) as a chaperone that is induced during development. We also show that HSP48 forms a biomolecular condensate and is stabilized by polyphosphate. The findings here identify Dictyostelium discoideum as a novel microbe to investigate protein quality control pathways during the transition to dormancy.

## INTRODUCTION

Upon cellular stress, cells elicit a number of responses, such as the induction of stress responsive genes, reduced translation, formation of phase-separated compartments, including stress granules, and the production of the chemical chaperone polyphosphate (1–10). Activation of these pathways provides defense mechanisms for cells to combat protein aggregation and promote cell survival. In addition to activating stress pathways upon experiencing cellular stress, some organisms enter dormancy, allowing them to survive adverse conditions, including extreme temperatures, desiccation, and starvation (11, 12).

One organism that enters a dormant state is the social amoeba Dictyostelium discoideum. Under conditions of cellular stress, D. discoideum undergoes a developmental process transitioning from a single cellular amoeba to a multicellular fruiting body containing dormant spores (13, 14). D. discoideum spores, which consist of a thick cellulose-rich spore encapsulating a dehydrated amoeba, can withstand unfavorable conditions such as extreme temperatures and desiccation (11). This developmental process allows D. discoideum to survive until environmental conditions have improved and the spores can germinate into amoeba (15).

One largely unexplored question is how proteostasis is maintained upon entry into dormancy. This is particularly interesting in the case of D. discoideum, as it encodes a proteome that contains a large number of repetitive amino acid tracts that are known to aggregate in other model organisms (16–18). Molecular chaperones, including the ATP-dependent foldases and the α-crystallin domain-containing holdases, are often upregulated upon cellular stress and play a protective role in maintaining proteostasis (19–21). Though it is known that D. discoideum harbors a large number of α-crystallin domain-containing proteins, their function in D. discoideum remains largely unexplored (16).

In addition to protein quality control pathways, the formation of stress-triggered biomolecular condensates also plays a protective role during cellular stress (2, 22–25). Biomolecular condensates are highly spherical membraneless compartments that comprise a variety of biological processes ranging from nucleolus formation to cellular signaling (26–32). Proteins that phase separate to form biomolecular condensates often contain intrinsically disordered domains that can enable liquid-liquid phase separation (5, 6, 27, 29, 33–36). In addition to intrinsically disordered domains, highly charged regions of proteins also drive phase separation via complementary electrostatic interactions (5, 37–41).

While little is known about stress responses during D. discoideum development, one potential chaperone that is increased during development is the chemical chaperone polyphosphate (10, 42). Polyphosphate plays a number of important roles in D. discoideum development, including regulating metabolism, spore germination, and fitness (10). In addition to its intracellular effects on D. discoideum development, excreted polyphosphate also promotes development by inhibiting proliferation and signaling through Ras and Akt (8, 9). In other model organisms, polyphosphate functions as a chaperone promoting the productive refolding of misfolded proteins and stabilizing amyloid fibers (43, 44).

How D. discoideum’s proteostatic network maintains proteostasis during its developmental cycle is largely unexplored. Here, we identify an uncharacterized α-crystallin domain-containing protein we named heat shock protein 48 (HSP48) whose transcript level is greatly induced during D. discoideum development. In cells, HSP48 undergoes liquid-liquid phase separation to form a biomolecular condensate in cells. In addition to HSP48’s N-terminal α-crystallin domain, HSP48 also contains a highly positively charged C terminus that is required for phase separation. In addition to HSP48 transcript, levels of the highly negatively charged primordial chaperone polyphosphate are also sharply increased during D. discoideum development, and polyphosphate is necessary to stabilize HSP48 protein levels. Upon germination, both HSP48 transcript and polyphosphate levels are rapidly diminished. Together, our data are consistent with a coordinated role for the molecular chaperone HSP48 and the chemical chaperone polyphosphate during D. discoideum development.

## RESULTS

### HSP48 is highly expressed during D. discoideum development.

**FIG 1:**
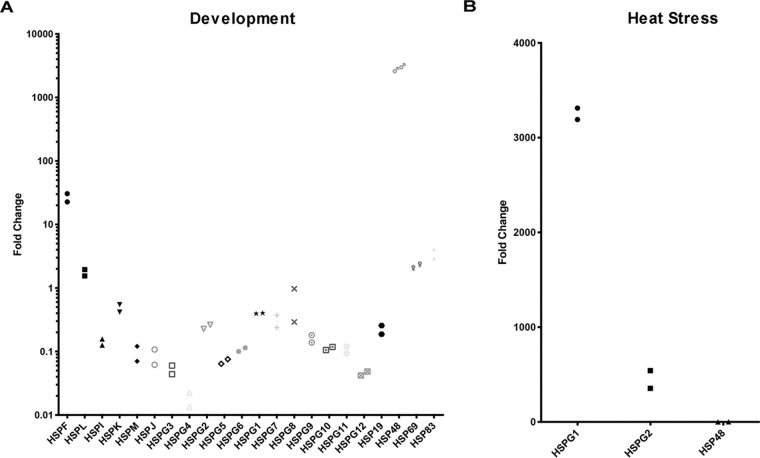
HSP48 is upregulated during Dictyostelium discoideum development. (A) HSP48 expression is upregulated during *Dictyostelium* development. Wild-type *Dictyostelium* cells were starved to induce development. Cells were harvested at 0 and 24 h for RNA extraction, and RT-PCR was performed to assess expression levels of α-crystallin domain-containing proteins (*n* = 2). (B) HSP48 is not induced upon heat stress. Wild-type *Dictyostelium* cells were grown at either 22°C or 30°C for 1 h and then harvested for RNA extraction. RT-PCR was performed to assess expression levels of the α-crystallin domain-containing proteins HSPG1, HSPG2, and HSP48 (*n* = 2).

We previously discovered that D. discoideum contains a large number of α-crystallin domain-containing proteins, including four previously unidentified α-crystallin domain-containing proteins (Table 1) (16). α-Crystallin domain-containing proteins have been implicated in the developmental process of some bacteria, leading us to hypothesize that they may also participate in D. discoideum development (45, 46). To begin assessing protein quality control pathways in D. discoideum development, we measured the expression levels of α-crystallin domain-containing proteins in the amoeba and upon completion of development. We identified one uncharacterized α-crystallin domain-containing protein, HSP48, whose transcript levels were induced greater than 1,000-fold in fruiting bodies compared to that in the amoeba (Fig. 1A). Unlike most other α-crystallin domain-containing proteins, such as HSPG1 and HSPG2, HSP48 expression did not increase upon heat stress, suggesting a more selective role in development (Fig. 1B). This suggests that HSP48 may play an important role in D. discoideum development.

**TABLE 1:** Newly identified α-crystallin-domain containing proteins

Gene name	Gene ID^*a*^
*hsp19*	DDB_G0295803
*hsp48*	DDB_G0280215
*hsp69*	DDB_G0283911
*hsp83*	DDB_G0288861

a ID, identifier.

### HSP48 forms a biomolecular condensate *in vivo*.

**FIG 2:**
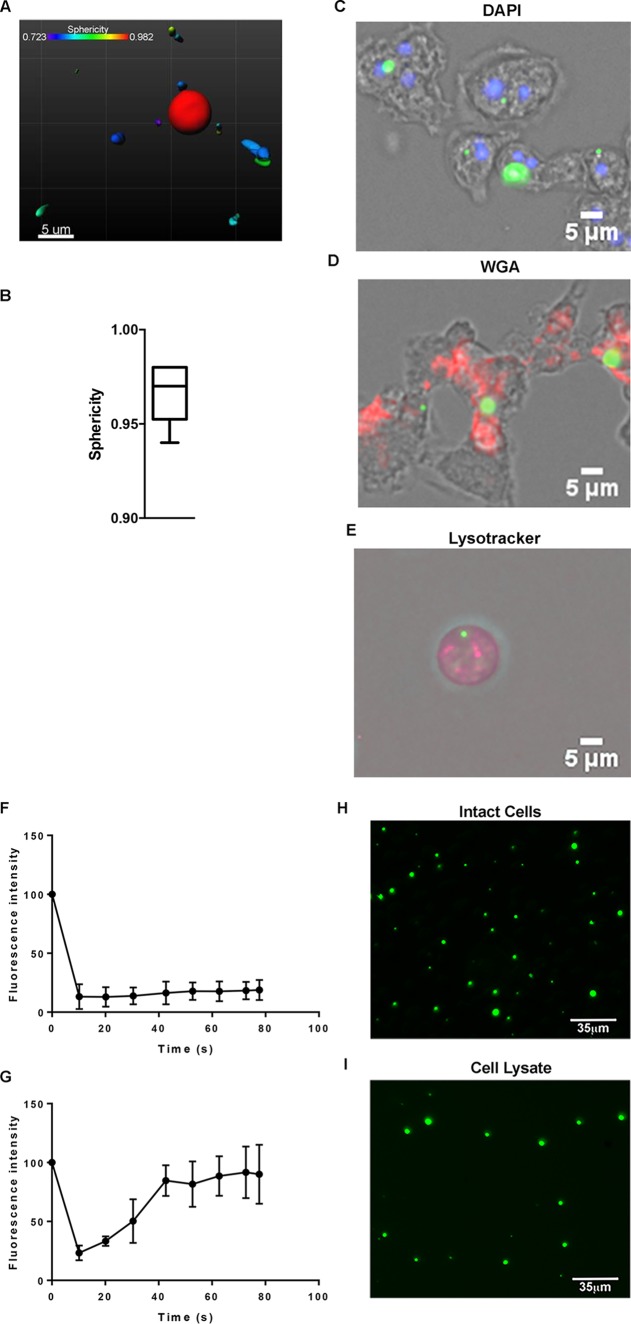
HSP48 phase separates to form a biomolecular condensate. (A) 3D volume-rendered HSP48. *Dictyostelium* cells expressing ^GFP^HSP48 were imaged using a confocal microscope. 3D volumes were then produced using Imaris software. (B) HSP48 puncta are highly spherical. 3D volume images of HSP48 were used to obtain sphericity values using Imaris software. Values equal to 1 indicate sphericity (*n* = 20). (C) HSP48 colocalizes to defined puncta. *Dictyostelium* cells were electroporated with a plasmid that expresses ^GFP^HSP48 and selected for a minimum of 2 weeks. Cells were fixed with methanol, stained with DAPI, and imaged with a fluorescence microscope (*n* = 3). (D) HSP48 colocalizes to defined puncta. *Dictyostelium* cells were electroporated with a plasmid that expresses ^GFP^HSP48 and selected for a minimum of 2 weeks. Cells were fixed with methanol, stained with wheat germ agglutinin (WGA), and imaged with a fluorescence microscope (*n* = 3). (E) HSP48 colocalizes to defined puncta. *Dictyostelium* cells were electroporated with a plasmid that expresses ^GFP^HSP48 and selected for a minimum of 2 weeks. Cells were stained with LysoTracker and imaged live with a fluorescence microscope (*n* = 3). (F and G) HSP48 FRAP analysis reveals two populations with different inherent mobilities. *Dictyostelium* cells were electroporated with a plasmid that expresses ^GFP^HSP48, selected for a minimum of 2 weeks, and used for FRAP. For FRAP, half of each individual droplet was bleached and then imaged for the indicated time points. ImageJ was then used for analysis to obtain fluorescence intensity values (*n* = 18). HSP48 puncta do not require a lipid bilayer. *Dictyostelium* cells were electroporated with a plasmid that expresses ^GFP^HSP48 and selected for a minimum of 2 weeks. Cells were either imaged as intact cells (H) or lysed (I) and imaged by fluorescence microscopy (*n* = 5).

Molecular chaperones can exist in various cellular compartments, including the endoplasmic reticulum, the nucleus, the cytoplasm, and the spore coat of developing bacteria (45–47). To determine HSP48’s cellular localization, we generated a green fluorescent protein (GFP)-tagged HSP48 construct (^GFP^HSP48) and expressed it in D. discoideum cells. ^GFP^HSP48 was present in the cytosol; however, rather than being diffuse, it formed puncta in cells (Fig. 2A). To assess the sphericity of ^GFP^HSP48 puncta, we obtained z-stacks of ^GFP^HSP48 puncta and analyzed three-dimensional (3D) reconstituted images using Imaris software (Fig. 2A). Consistent with HSP48 forming nearly perfect spheres, the mean average sphericity of ^GFP^HSP48 was calculated to be >0.95 (Fig. 2B). The ^GFP^HSP48 puncta did not appear to colocalize with the nucleus, lysosome, or *trans*-Golgi (Fig. 2C to E), suggesting that HSP48 may undergo liquid-liquid phase separation. Because protein aggregates may also form spherical structures, we next wanted to measure the dynamics of HSP48 spheres. Unlike protein aggregates where proteins are highly immobile in a solid-like state, biomolecular condensates are present in both highly dynamic liquid-like states and more glass-like solid states (26). To determine if HSP48 was in either a liquid or solid state, we next performed fluorescence recovery after photobleaching (FRAP) analysis on ^GFP^HSP48 puncta. In our FRAP analysis, half of a ^GFP^HSP48 biomolecular condensate was bleached, and recovery of fluorescence in the bleached area was then measured over time. Similarly to other biomolecular condensates, we observed multiple states for ^GFP^HSP48 with one population (∼72%) behaving more solid like (less mobility within the condensate) (Fig. 2F) and a second population (∼28%) behaving more liquid like (more mobility within the condensate) (Fig. 2G) (48, 49). Additionally, unlike membrane-bound organelles and vesicles, biomolecular condensates form compartments that do not require a lipid bilayer (4, 29, 50). To determine if HSP48 puncta formation required a lipid bilayer, we disrupted lipid bilayers with detergent and looked to see if ^GFP^HSP48 remained as puncta in the cell lysates. Consistent with HSP48 puncta being a membraneless structure, ^GFP^HSP48 puncta remained after cell lysis with detergent (Fig. 2H and I). Together, these data suggest that HSP48 forms a biomolecular condensate in the cytoplasm of D. discoideum.

### HSP48’s intrinsically disordered C-terminal region is necessary for biomolecular condensate formation.

**FIG 3:**
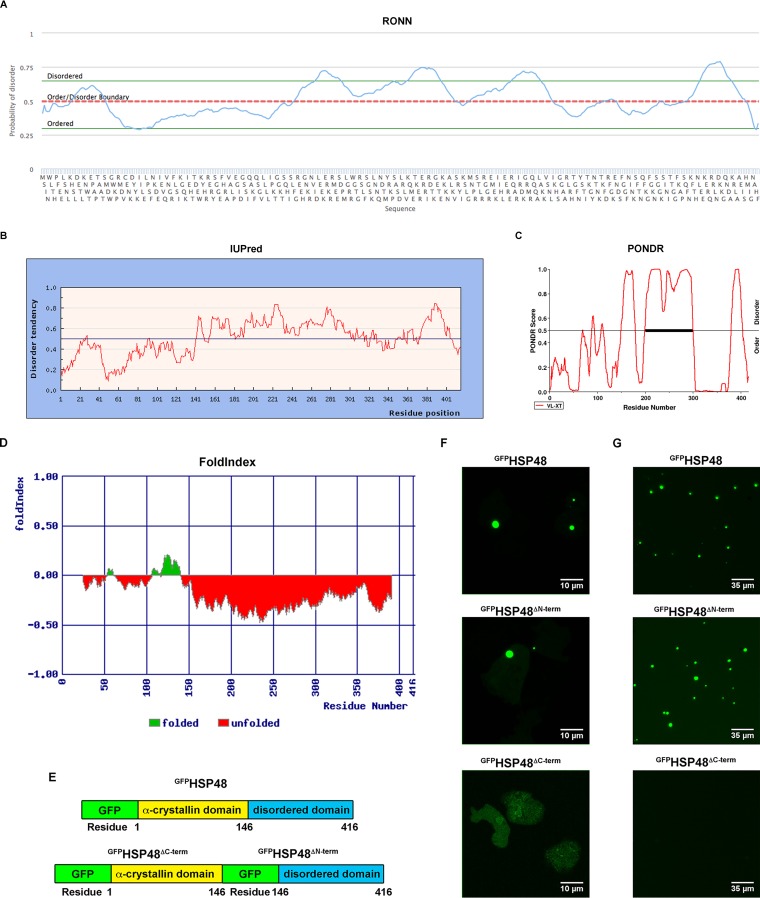
A region next to HSP48’s C terminus drives phase separation. A region next to HSP48’s C terminus is intrinsically disordered. HSP48’s amino acid sequence was analyzed for its probability of disorder using RONN (A), IUPred (B), PONDR (C), and FoldIndex (D). (E and F) HSP48’s intrinsically disordered region is necessary for liquid droplet formation. *Dictyostelium* cells were electroporated with plasmids that express either ^GFP^HSP48, ^GFP^HSP48^ΔN-term^, or ^GFP^HSP48^ΔC-term^ and selected for a minimum of 2 weeks. Cells were then imaged using confocal microscopy (*n* = 3). (G) HSP48 puncta do not require a lipid bilayer. *Dictyostelium* cells were electroporated with plasmids that express either ^GFP^HSP48, ^GFP^HSP48^ΔN-term^, or ^GFP^HSP48^ΔC-term^ and selected for a minimum of 2 weeks. Cells were then lysed and imaged using a fluorescence microscope (*n* = 5).

One common characteristic of proteins that form biomolecular condensates is the presence of an intrinsically disordered domain (5, 6, 27–29, 31, 37, 38, 51–54). To determine if HSP48 has an intrinsically disordered domain, we performed *in silico* analysis of HSP48’s sequence using multiple algorithms, including RONN, PONDR, FoldIndex, and IUPred. With each algorithm, a region next to the C terminus of HSP48 was predicted to be intrinsically disordered (Fig. 3A to D). We next wanted to determine if HSP48’s intrinsically disordered domain is responsible for driving its phase separation. To accomplish this, we made HSP48 constructs with deletions of either the C-terminal intrinsically disordered domain (^GFP^HSP48^ΔC-term^) or HSP48’s N-terminal α-crystallin domain (^GFP^HSP48^ΔN-term^) and tested their ability to form biomolecular condensates in cells (Fig. 3E). Consistent with a region next to HSP48’s C terminus driving phase separation, ^GFP^HSP48^ΔC-term^ did not form a biomolecular condensate but instead was diffuse, while both wild-type ^GFP^HSP48 and the ^GFP^HSP48^ΔN-term^ retained their ability to form puncta in cells (Fig. 3F). Consistent with our *in vivo* data, ^GFP^HSP48 and ^GFP^HSP48^ΔN-term^ puncta remained after cell lysis, while ^GFP^HSP48^ΔC-term^ was diffuse (Fig. 3G). Together, these data demonstrate that the formation of an HSP48 biomolecular condensate is driven by the region next to its C terminus.

### Polyphosphate regulates HSP48 phase separation and stability.

**FIG 4:**
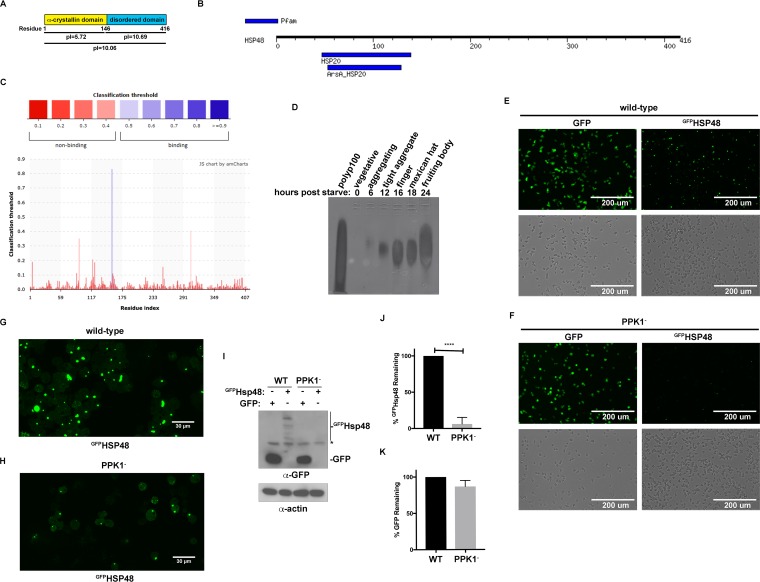
Polyphosphate drives HSP48 phase separation and stabilization. (A) HSP48 has a highly basic C terminus. *In silico* analysis of HSP48 was performed to determine the isoelectric point. (B) HSP48 is not predicted to have a DNA or RNA binding motif. HSP48’s amino acid sequence was analyzed by MOTIF search against the Pfam database. (C) HSP48 is not predicted to bind RNA. HSP48’s amino acid sequence was analyzed by PRIdictor. (D) Polyphosphate levels increase during *Dictyostelium* development. Wild-type AX4 cells were developed and harvested at the indicated time points. To image polyphosphate, RNA was isolated, run on an acrylamide gel, and stained with DAPI (*n* = 3). (E, F) Polyphosphate promotes HSP48 phase separation *in vivo.* ^GFP^HSP48 constructs were transformed into wild-type (C) and △PPK1 (PPK1^−^) (D) cells. Cells were selected and then imaged using a fluorescence microscope (*n* = 4). (G) Confocal image of ^GFP^HSP48 puncta in wild-type cells. Wild-type cells electroporated with a plasmid that expresses ^GFP^HSP48 and were imaged by confocal microscopy (*n* = 4). (H) Confocal image of ^GFP^HSP48 puncta in △PPK1 cells. △PPK1 cells were electroporated with a plasmid that expresses ^GFP^HSP48 and were imaged by confocal microscopy (*n* = 4). (I) ^GFP^HSP48 protein levels are decreased in △PPK1 cells. Wild-type and △PPK1 cells expressing either ^GFP^HSP48 or GFP were analyzed by Western blotting (*n* = 3). *, nonspecific band. (J) ^GFP^HSP48 protein levels are decreased in △PPK1 cells. Quantification of HSP48 in wild-type and △PPK1 cells (*n* = 3). ****, *P* < 0.0001. (K) GFP protein levels are unchanged in △PPK1 cells. Quantification of GFP in wild-type and △PPK1 cells (*n* = 3).

We next wanted to understand how this region next to HSP48’s C terminus drives biomolecular condensate formation. Previous work demonstrated that complementary electrostatic interactions drive the formation of many biomolecular condensates (5, 37–41), and so we analyzed the region next to HSP48’s C terminus to identify charged regions. Using *in silico* analysis, we found that the intrinsically disordered region next to HSP48’s C terminus is highly basic with an isoelectric point of 10.69, while HSP48’s N-terminal α-crystallin domain has an isoelectric point of 5.72 (Fig. 4A). One common negatively charged molecule that drives the formation of biomolecular condensates is RNA (5, 6, 28, 29, 36, 37, 52, 55). To determine if HSP48 contained any predicted RNA-binding motifs, we next performed an *in silico* analysis using MOTIF and PRIdictor (56, 57); however, neither program identified an RNA-binding domain within HSP48 (Fig. 4B and C).

Because HSP48 lacked a predicted RNA-binding motif, we hypothesized another highly acidic molecule may be driving HSP48 phase separation. Because HSP48 accumulates during development, we turned our attention to polyphosphate, a highly negatively charged molecule whose levels dramatically increase during D. discoideum development (10). To confirm that polyphosphate levels increase during D. discoideum development, we induced D. discoideum development by starvation and analyzed polyphosphate accumulation at 0, 6, 12, 16, 18, and 24 h by using an established technique (10). Consistent with previous studies, we found that polyphosphate levels increased during D. discoideum development similarly to HSP48 (10) (Fig. 4D). To determine if polyphosphate was necessary for HSP48 phase separation, we expressed ^GFP^HSP48 in D. discoideum cells lacking polyphosphate kinase 1 (PPK1), the major enzyme responsible for polyphosphate production in D. discoideum (10). In ΔPPK1 cells, the size and number of the ^GFP^HSP48 biomolecular condensates formed were dramatically decreased (Fig. 4E to H). In addition to decreasing ^GFP^HSP48 puncta, we also observed a decrease in diffuse ^GFP^HSP48, suggesting that ^GFP^HSP48 is destabilized in the absence of polyphosphate (Fig. 4E to H). Western blot analysis of wild-type and ΔPPK1 cells expressing ^GFP^HSP48 verified that while the GFP control remained unchanged, ^GFP^HSP48 levels were decreased in ΔPPK1 cells, suggesting a potential role for polyphosphate in regulating HSP48 protein levels (Fig. 4I). Because polyphosphate plays a role in regulating transcription and translation (58–60), we wanted to confirm that ^GFP^HSP48 destabilization was not due to differences in transcription and translation. Consistent with no change in HSP48 transcription or translation, fluorescence levels were similar in wild-type and ΔPPK1 cells expressing GFP alone, indicating that global transcription and translation were not altered (Fig. 4E to K). Because transcription and translation were unaffected, this suggested that polyphosphate may be functioning to stabilize HSP48 by preventing its degradation. Together, these data are consistent with a role for polyphosphate in stabilizing ^GFP^HSP48 by preventing its clearance.

**FIG 5:**
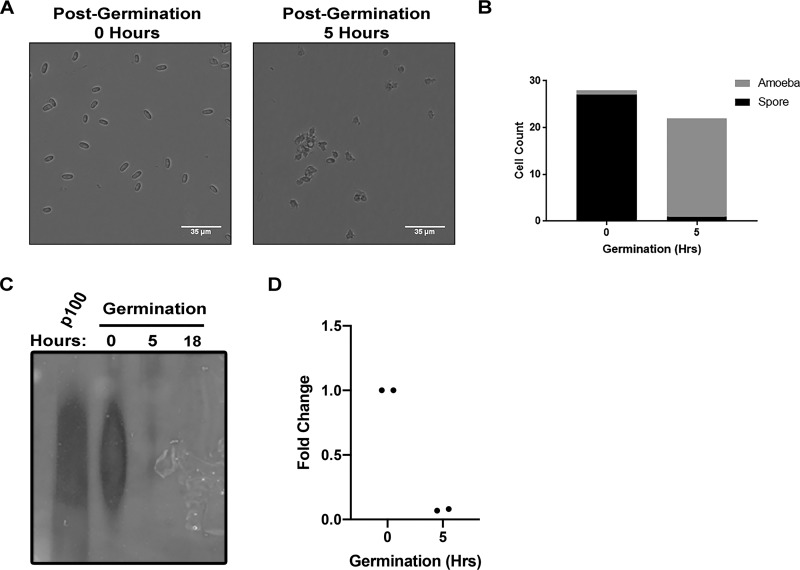
Polyphosphate and HSP48 levels dramatically decrease upon germination. (A) Wild-type AX4 cells were developed for 24 h, after which, spores were isolated and cultured in medium for the indicated time points. Cells were then imaged using bright-field microscopy. (B) Quantification of the numbers of amoeba and spores at 0 h (number of cells counted = 28) and 5 h postgermination (number of cells counted = 22). (C) Polyphosphate decreases rapidly after germination. Wild-type AX4 cells were developed for 24 h, after which, spores were isolated and cultured in medium for the indicated time points. RNA was extracted to obtain polyphosphate, run on an acrylamide gel, and stained with DAPI (*n* = 3). (D) HSP48 transcript levels decrease rapidly after germination. Wild-type AX4 cells were developed for 24 h, after which, spores were isolated and cultured in medium for the indicated time points. RNA was extracted, and RT-PCR was performed to assess expression levels of HSP48 (*n* = 3).

We observed that transcript levels for HSP48 were dramatically increased upon D. discoideum development (Fig. 1A), and we and others have observed that polyphosphate levels are robustly upregulated during D. discoideum development (Fig. 4D) (10). We next wanted to determine if polyphosphate and HSP48 transcript levels are downregulated upon germination of D. discoideum spores. To accomplish this, we analyzed D. discoideum spores and amoeba that had been allowed to germinate for 5 h, a time point where greater than 95% of spores had germinated (Fig. 5A and B). Consistent with polyphosphate levels dramatically decreasing upon germination, polyphosphate was undetectable 5 h after the addition of nutrients (Fig. 5C). We next wanted to determine if HSP48 transcript levels also decreased upon germination. To determine this, we assessed HSP48 transcript levels in spores and amoeba 5 h after induction of germination. Consistent with HSP48 transcript levels being downregulated upon germination, we detected nearly no HSP48 transcript in germinated cells (Fig. 5D). Together, these data are consistent with a rapid downregulation of both HSP48 and polyphosphate upon germination of D. discoideum spores.

## DISCUSSION

Here, we identify HSP48 as a novel α-crystallin domain-containing protein that is vastly upregulated during D. discoideum development (Fig. 1). HSP48 forms a biomolecular condensate, a process dependent on its highly basic intrinsically disordered region next to the C terminus (Fig. 2 and 3). The highly acid chemical chaperone, polyphosphate, is necessary to stabilize HSP48, and deletion of PPK1, the major enzyme responsible for polyphosphate production in D. discoideum, results in a destabilization of HSP48 levels (Fig. 4). Finally, upon germination, levels of both HSP48 transcript and polyphosphate are dramatically decreased, consistent with their role in development (Fig. 5). Moving forward, it will be important to determine if HSP48 is essential for D. discoideum development and, if so, what role it plays.

How D. discoideum maintain proteostasis during their developmental process is largely unknown. However, it is known that protein degradation pathways are essential for D. discoideum development. Deletion of autophagy genes, including *atg1*, *atg5*, *atg6*, *atg7*, and *atg8*, or of the deubiquitinating enzyme UbpA results in abnormal development (61–63). Similarly, the molecular chaperone Hsp90 is also necessary for D. discoideum development, as Hsp90 inhibition with geldanamycin arrests D. discoideum development at the mound stage (64). However, it is unclear if the defects in development result from a broad disruption in proteostasis or from a disruption of select pathways. While we observed a large induction of HSP48 during D. discoideum development (Fig. 1), levels of another α-crystallin domain-containing protein, HSP32, decreased, suggesting that components of the proteostatic network may be tightly regulated during D. discoideum development (65). Because HSP48 is an α-crystallin domain-containing protein, it will be important to identify its role in maintaining proteostasis during D. discoideum development. Moving forward, it will be interesting to identify other protein quality control components that help maintain proteostasis during development. This will be particularly interesting in D. discoideum spores, as their highly compact desiccated environment presents unique challenges in maintaining proteostasis.

Though D. discoideum development provides a unique model for interrogating protein quality control pathways, HSP48 also provides a potential model for studying phase separation. In recent years, phase separation has been identified as a mechanism to regulate transcription and protein aggregation under various conditions (25, 66–68). Stress granules, which have become the primary system for studying membraneless organelles and phase separation, have been greatly associated with neurodegenerative diseases, as an increasing number of disease-associated proteins have been shown to phase separate (6, 7, 26, 28, 36, 41, 69–72). For instance, the identification of a liquid-to-solid-phase transition with disease-associated FUS mutations led to the recognition of aberrant phase separation as a major contributing factor in amyotrophic lateral sclerosis (ALS) (28, 36, 41). Additionally, the phase separation of individual proteins has now been tied back to other major diseases, such as the polyglutamine diseases and Alzheimer’s disease (39, 40, 52, 69, 73, 74). Interestingly, the resulting solid states of phase-separated proteins greatly resemble protein fibrils seen in disease. However, it is unclear whether phase separation functions as a mechanism for amyloid fibril formation or whether it functions as an independent pathway. A greater understanding of this process is necessary for the development of therapeutics for neurodegenerative diseases associated with aberrant phase separation.

While aberrant phase separation is observed in many neurodegenerative diseases, it is also becoming clear that phase separation serves an important function during cellular stress (2, 23). For example, stress granules in response to various stressors often serve to increase cellular fitness, suggesting that phase separation may serve as a general stress response (22, 23). In a similar stress response, cells may enter a highly desiccated dormant state in which the cytosol phase transitions from a liquid to a glass-like state (12, 24, 75–77), slowing down dynamics within the cell. In this manner, phase separation may serve as a protective effect providing sufficient time for the sequestration and refolding of misfolded proteins (78). In line with this, the small heat shock protein Hsp42 utilizes a prion-like domain to sequester misfolded proteins and utilizes a second intrinsically disordered domain to regulate this process. Interestingly, removal of the second intrinsically disordered domain improves the chaperone function of Hsp42; however, both domains are needed to promote cellular fitness, suggesting that regulating the sequestration of misfolded proteins is equally important (66). We speculate that HSP48 may similarly phase separate and bind misfolded proteins, allowing HSP48 to sequester proteins and maintain their solubility during D. discoideum development. This raises the possibility for phase separation to serve as a stress response that functions to sequester proteins and maintain proteostasis.

Previous work has shown that electrostatic interactions between RNA and protein can induce phase separation. For example, stress granules form in part via electrostatic interactions between negatively charged RNA and positively charged regions of RNA-binding proteins (6). Here, we show that polyphosphate, a highly negatively charged molecule whose levels vastly increase during D. discoideum development, promotes HSP48’s phase separation (Fig. 4). The formation of a polyphosphate-HSP48 biomolecular condensate raises the possibility that HSP48 may act as a chaperone in multiple ways. On the one hand, polyphosphate is a chemical chaperone that binds unfolded proteins and supports productive refolding upon resolution of cellular stress (44). HSP48’s α-crystallin domain coupled with polyphosphate’s role as a chemical chaperone may act as a chaperoning complex that helps maintain proteostasis during development. Alternatively, polyphosphate promotes the conversion of misfolded proteins to form amyloid fibrils (43). In this regard, HSP48 may sequester polyphosphate in a biomolecular condensate, thus preventing it from promoting the formation of amyloid during D. discoideum development. In the future, D. discoideum development may serve as a useful model organism to determine whether polyphosphate is protective or detrimental in diseases of protein misfolding.

In addition to preventing polyphosphate-induced aggregation, the formation of an HSP48 biomolecular condensate may also serve as storage mechanism for polyphosphate during development. In other organisms, membrane-bound organelles known as acidocalcisomes have been observed to store calcium and polyphosphate (79). More recently, polyphosphate (polyP) storage granules lacking a membrane were identified in Acetonema longum during sporulation (80). In D. discoideum, as cells enter dormancy, an HSP48 biomolecular condensate may serve as a membraneless compartment for polyphosphate storage, providing a mechanism by which metabolism may be regulated. In this manner, polyphosphate would be sequestered in spores as a readily available energy source upon germination. In addition to D. discoideum, a number of microbes undergo a developmental process during cellular stress leading to the formation of dormant spores. As polyphosphate storage has been implicated for survival in response to various stressors, it would be interesting to see if phase separation serves as the main mechanism for sequestering polyphosphate, providing an additional avenue for targeting drug-resistant microbes.

## MATERIALS AND METHODS

### Expression constructs and antibodies.

HSP48 (DDB_G0280215; XP_641311.1) was PCR amplified from D. discoideum cDNA and cloned into Pet28 and pTxGFP (Dictybase) using BamHI and XhoI. Anti-ubiquitin antibody was from Invitrogen (14-6078-82) and anti-GFP was from Life Technologies (A11122). Peroxidase-conjugated secondary antibodies were from Jackson ImmunoResearch. IgG-fluorescein isothiocyanate (FITC) secondary was used for immunostaining (sc-2010; Santa Cruz). β-Actin (PA121167; Pierce) was used as a loading control.

### Dictyostelium discoideum cell culture and transformations.

Dictyostelium discoideum AX4 cells were maintained in shaking cultures at 22°C in HL5 medium. Cells were subcultured to maintain a density no greater than 6 × 10^6^ cells/ml.

Transformations were performed as previously described (81). Briefly, 5 × 10^6^ cells were washed with H-50 buffer (20 mM HEPES, 50 mM KCl, 10 mM NaCl, 1 mM MgSO_4_, 5 mM NaHCO_3_, 1 mM NaH_2_PO_4_; pH adjusted to 7.0 with HCl/NaOH). Cells were electroporated in a 1-mm cuvette (0.85 kV/25 μF, 0.6 ms, twice with 5 s in between). Cells were selected with 10-μg/ml G-418 for 1 week (82).

### Development.

To induce development, 2 × 10^8^ cells were washed and grown on filter paper soaked with filtered developmental buffer (5 mM Na_2_HPO_4_, 5 mM KH_2_PO_4_, 1 mM CaCl_2_, 2 mM MgCl_2_; pH adjusted to 6.5 with HCl/NaOH) at 22°C (14). Cells were harvested at various time points for RNA isolation.

For microscopy, 2 × 10^8^ cells were washed with development buffer and grown on KK2 plates (14). Cells were developed for various time points and imaged using a Leica MZFL3 fluorescence stereomicroscope.

### Heat stress.

Briefly, 1 × 10^7^ cells were incubated in HL5 medium at a density of 1 × 10^6^ cells/ml at either 22°C or 30°C for 1 h. Cells were then harvested and used for RNA isolation.

### RNA isolation and cDNA synthesis.

For RNA isolation, Qiagen’s RNeasy Mini kit was used according to the manufacturer’s instructions. An optional DNase digestion step was performed, and the elution step was performed with RNase-free water. Invitrogen’s SuperScript III First-Strand Synthesis System for real-time PCR (RT-PCR) was used for cDNA synthesis according to the manufacturer’s instructions.

### RT-PCR.

Real-time PCR was performed using iQ SYBR green supermix (170-8880; Bio-Rad) in 96-well plates (Pryme PCR, AVRT1; MidSci) sealed with adhesive seals (MSB1001; Bio-Rad) in an Eppendorf RealPlex^2^. Data were analyzed using the Livak method.

### Immunocytochemistry.

Cells were harvested at various time points and plated on 24-well plates at a density of 7.5 × 10^5^ cells/ml. Cells were allowed to settle and then fixed with cold 100% methanol for 10 min at −20°C. Cells were briefly washed to remove remaining methanol and incubated in blocking buffer (2% bovine serum albumin [BSA], 1% Triton X-100 in 1× phosphate-buffered saline [PBS]) for 30 min at room temperature. Cells were then incubated in primary antibody (1:2,000 in blocking buffer) overnight at 4°C. Cells were washed three times with PBT (0.1% Triton X-100, 0.5% BSA in 1× PBS) and then incubated in secondary (1:500 in blocking buffer) for 2 h at room temperature in the dark. Cells were washed three times with PBT and imaged using an Evos FL Auto microscope (Life Technologies).

### Confocal microscopy.

Briefly, 3 × 10^6^ cells were plated on coverslips, allowed to settle, and then fixed with cold 100% methanol for 10 min at −20°C. Cells were washed and stained with DAPI (4′,6-diamidino-2-phenylindole; Life Technologies). Coverslips were mounted with ProLong Gold Antifade reagent (Life Technologies) and imaged with a Leica TCS Sp5 confocal scope. Z-stack images were captured at ×63 magnification at 0.5-μm intervals and 1,024 × 1,024 pixel resolution and merged using Fiji.

For live cell imaging, D. discoideum cells were plated on 2% low-melt agarose pads in starvation buffer. Slides were prepared as previously described. Cells were imaged with a Nikon Eclipse 90i confocal microscope at ×100 magnification. Z-stack images were obtained at 0.5-μm intervals and 1,024 × 1,024 pixel resolution and merged using Fiji.

### Fluorescence microscopy.

For live cell imaging, cells were washed with starvation buffer (0.1 M MES [morpholineethanesulfonic acid], 0.2 mM CaCl_2_, 2 mM MgSO_4_, pH adjusted to 6.8 with HCl/NaOH) and imaged at ×20 magnification using an Evos FL Auto microscope (Life Technologies) (83). For lysate imaging, 1 × 10^7^ cells were lysed with NETN (0.5% Nonidet P-40, 150 mM NaCl, 50 mM Tris, and protease inhibitors [Roche Applied Science]) and sonicated using an ultrasonic cell disruptor 2 times for 10 s on ice at 30% output. Lysis was then confirmed by bright-field imaging before imaging fluorescence at ×20 magnification using an Evos FL Auto microscope (Life Technologies).

### Spore germination.

AX4 cells were developed for 2 to 3 days. For spore isolation, developed cells were harvested, resuspended in 1 ml of 10 mM MES (pH adjusted to 6.5 with HCl or NaOH), and vortexed for 1 min. An additional 4 ml of buffer was added to cells and they were vortexed again for 1 min. Cell suspensions were passed through a 114 Whatman filter paper (84). Spores were then resuspended at a density of 1.5 × 10^6^ cells/ml in HL5 medium to induce germination and harvested for RNA isolation at various time points (10).

### Polyphosphate gels.

Polyphosphates gels (15% polyacrylamide [1610154; Bio-Rad], 7 M urea in Tris-borate-EDTA [TBE], 10% ammonium persulfate [APS], *N*,*N*,*N*′,*N*′-tetramethylethylenediamine [TEMED]) were prerun for 30 min at 100 V with running buffer (89 mM Tris, 89 mM borate, 2 mM EDTA, pH 8.3). Wells were then rinsed with running buffer. Five micrograms of RNA for each sample was prepared with 6× loading dye (0.01% bromophenol blue, 30% glycerol, 10 mM Tris-HCl [pH 7.4], 1 mM EDTA), loaded, and run on a polyphosphate gel at 150 V. PolyP 100 was used as a positive control. Gels were next rocked in staining solution (20% methanol, 2% glycerol, 20 mM Tris base, 2 μg/ml DAPI) at room temperature for 30 min), followed by incubation in destain solution (20% methanol, 2% glycerol, 20 mM Tris base) for 45 min (10). For images, gels were exposed at 365 nm using an EpiChemi^3^ Darkroom system (UVP Bioimaging Systems).

### Western blotting.

For Western blotting, protein samples were prepared by lysing 1 × 10^7^ cells with NETN (0.5% Nonidet P-40, 150 mM NaCl, 50 mM Tris, and protease inhibitors [Roche Applied Science]), followed by sonication using an ultrasonic cell disruptor two times for 10 s on ice at 30% output. Protein concentration was determined by a bicinchoninic acid (BCA) assay, and then 10 μg of protein was run on SDS-PAGE gels and transferred to a polyvinylidene difluoride (PVDF) membrane. Membranes were blocked with 5% milk in TBST (Tris-buffered saline, 0.1% Tween 20) and put in primary antibody at 1:1,000 overnight at 4°C. Secondary antibody was used at 1:5,000 at room temperature for 1 h.

### Fluorescence recovery after photobleaching.

FRAP analysis was performed as previously described (85). Briefly, a fluorescence recovery after photobleaching (FRAP) wizard on a Leica TCS Sp5 was used for photobleaching and recovery imaging. Imaging was performed at ×63 magnification, and the region of interest was selected as half of a droplet. Bleaching was performed at 100% for 40 scans at 0.15 s. Baseline fluorescence and fluorescence recovery parameters were set as recommended by the FRAP wizard manual. Fluorescence intensity was analyzed using Fiji.

### Sphericity.

Z-stack images were used to render a 3D volume image using Imaris Software. Imaris surface creation wizard was then used to create a model of the 3D volume. A Gaussian filter and background subtraction were applied to improve the algorithm. Sphericity was then obtained using the statistics section in Imaris. Sphericity values were then plotted using GraphPad Prism.

### Statistics.

Statistics were performed using GraphPad Prism 8 software. Comparisons were made using a two-tailed *t* test for Fig. 4H and I. Error bars are standard deviations of the means. In all figure legends, *n* refers to the number of independent experiments performed.
